# In-vacuum long-wavelength macromolecular crystallography

**DOI:** 10.1107/S2059798316001078

**Published:** 2016-03-01

**Authors:** Armin Wagner, Ramona Duman, Keith Henderson, Vitaliy Mykhaylyk

**Affiliations:** a Diamond Light Source, Harwell Science and Innovation Campus, Chilton, Didcot OX11 0DE, England

**Keywords:** long-wavelength X-rays, soft X-rays, native phasing, S-SAD, synchrotron beamline

## Abstract

Structure solution based on the weak anomalous signal from native (protein and DNA) crystals is increasingly being attempted as part of synchrotron experiments. Maximizing the measurable anomalous signal by collecting diffraction data at longer wavelengths presents a series of technical challenges caused by the increased absorption of X-rays and larger diffraction angles. A new beamline at Diamond Light Source has been built specifically for collecting data at wavelengths beyond the capability of other synchrotron macromolecular crystallography beamlines. Here, the theoretical considerations in support of the long-wavelength beamline are outlined and the in-vacuum design of the endstation is discussed, as well as other hardware features aimed at enhancing the accuracy of the diffraction data. The first commissioning results, representing the first in-vacuum protein structure solution, demonstrate the promising potential of the beamline.

## Abstract 2

The motivation for, and the first results from, the novel in-vacuum long-wavelength MX beamline I23 at Diamond Light Source are presented.

## 1. Introduction   

Long-wavelength macromolecular crystallography (MX) offers direct access to solving the crystallographic phase problem through exploitation of the small anomalous differences from S and P atoms present in native protein and nucleic acid crystals without the need for additional heavy-atom labelling. The technique of single-anomalous diffraction experiments from sulfur (S-SAD) was first successfully demonstrated in 1981 for the small protein crambin (Hendrickson & Teeter, 1981 ▸). The idea of using S-SAD as a tool for solving protein structures was reintroduced in 1999 (Dauter *et al.*, 1999 ▸) and a first novel structure was published in 2000 (Liu *et al.*, 2000 ▸). However, the number of novel structures determined by native S-SAD or P-SAD has remained small. Only recently have several challenging crystal structures been determined employing multi-crystal averaging (Liu *et al.*, 2012 ▸; El Omari *et al.*, 2014 ▸) and low-dose experiments in multiple orientations from only one crystal (Weinert *et al.*, 2015 ▸). Aside from continuous successful efforts to reduce instrument errors introduced by the beamline hardware, the main contributing factors to these recent breakthroughs have been the introduction of pixel-array detectors (Broennimann *et al.*, 2006 ▸) and improved software for data processing and structure solution (Kabsch, 2010 ▸; Sheldrick, 2010 ▸; Adams *et al.*, 2010 ▸).

Native SAD experiments are not the only useful application of long-wavelength work. Tuning the wavelength below and above specific absorption edges can allow the unambiguous determination of bound cofactors such as Cl^−^, K^+^ or Ca^2+^. Model building can be assisted by the utilization of anomalous difference Fourier maps to locate the positions of sulfur to anchor the protein sequence into low-resolution electron-density maps from molecular-replacement solutions. Additionally, the large anomalous signal from the *M*_V_ edge of uranium (*f*′′ > 100 e^−^) can assist in the phasing of large macromolecular complexes by binding uranyl salts to the proteins (Liu *et al.*, 2001 ▸).

The design of MX beamlines at synchrotrons has been optimized over the past years towards fully automated high-throughput instruments for wavelengths around the Se *K* edge (λ = 0.979 Å). Many of these beamlines can access wavelengths up to 2.3 Å and most of the work cited above has been successfully performed in this regime (Rose *et al.*, 2015 ▸). Beamline BL-1A at the Photon Factory can access wavelengths up to 3.5 Å and the first successful results have been published (Ru *et al.*, 2012 ▸). Earlier attempts to access the sulfur and phosphorus *K* edges (λ = 5.015 and 5.779 Å, respectively) at HASYLAB (Stuhrmann *et al.*, 1997 ▸) and ESRF (Biou *et al.*, 2005 ▸) were promising, but were clearly limited by the technology available at the time. The most relevant factors to be considered to overcome the technical challenges for long-wavelength MX have been summarized by Djinovic Carugo *et al.* (2005 ▸). The design of the long-wavelength MX beamline I23 at Diamond Light Source (DLS) addresses all of these aspects and is described below.

## 2. Theoretical considerations   

### 2.1. Wavelength dependency of scattering and absorption   

Various physical processes occur during the interaction of X-rays with matter. These processes can be separated into elastic scattering in which no energy is lost, inelastic Compton scattering (with energy loss) and, most relevant to the present case, photo-absorption. In the last of these the incident radiation is absorbed, resulting in the ejection of an electron from an atom. This behaviour is element-specific and displays a strong dependence on the wavelength of the incident radiation, exhibiting so-called absorption edges. The scattering processes change rather more smoothly with wavelength.

**Figure 1:**
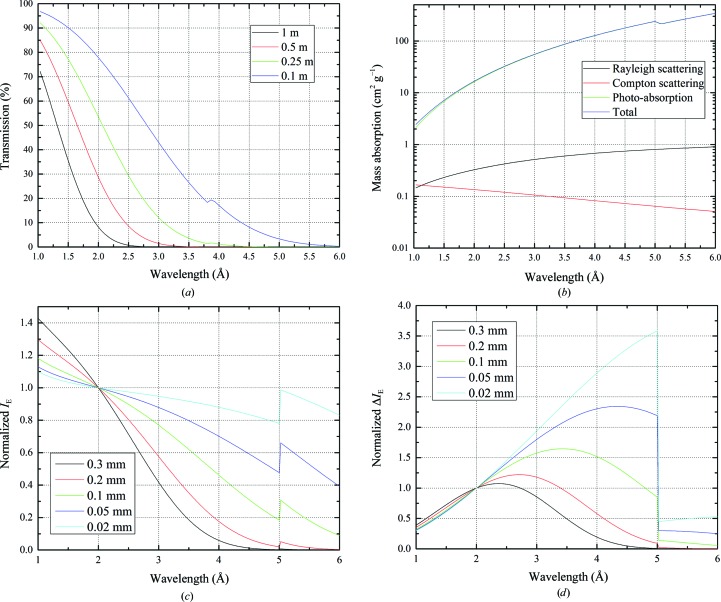
X-ray transmission through different lengths of air as a function of wavelength (*a*), mass attenuation coefficients for lysozyme (*b*) and normalized dose-weighted diffraction intensity *I*_E_ (*c*) and normalized dose-weighted anomalous diffraction intensity difference Δ*I*_E_ (*d*) for different sized isometric lysozyme crystals.

Scattering and absorption occur not only within the crystal itself, but also within any surrounding material and within both the incident and diffracted beam paths. For conventional beamlines in air at atmospheric pressure, the attenuation of long-wavelength radiation with distance becomes significant. Fig. 1 ▸(*a*) shows the transmission through several path lengths of air in the wavelength range from 1 to 6 Å. In addition to the weakening of the signal caused by this attenuation, X-ray scattering within the beam path gives rise to background noise on the detector, hence limiting the data quality. Such factors are greatly reduced by the use of a light gas, such as helium, or are in principle removed by performing the experiment in vacuum.

The reduction in the intensity of an incident beam is exponential with the thickness of the material through which the beam passes. This process can be described by a linear absorption coefficient μ_l_, which is characteristic of the chemical composition and density of the material. Microscopically, this can be understood by the superposition of individual atoms within the material through the concept of atomic cross-sections. The linear absorption coefficient can be expressed as a sum of the effects of the atoms of each element based on the chemical composition of the material, 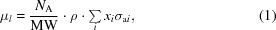
where *N*_A_ is Avogadro’s number, MW is the molecular weight, ρ is the density, *x*_*i*_ is the number of atoms of type *i* from the chemical formula and σ_a*i*_ is the absorption cross-section of the different elements *i*. Fig. 1 ▸(*b*) shows the relative contributions to the linear absorption coefficient of lysozyme for the different physical processes in the wavelength range from 1 to 6 Å as calculated by *XOP* (Sánchez del Río & Dejus, 2011 ▸). Photo-absorption dominates the linear attenuation of the material.

The imaginary part of the anomalous scattering factor is proportional to the absorption cross-section of an atom, as expressed by
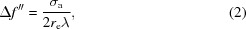
where *r*_e_ is the classical radius of the electron and λ is the wavelength.

### 2.2. Optimal wavelength for native SAD   

In the presence of heavy atoms, the choice of wavelength for MAD or SAD experiments at a tuneable X-ray source is at or around an accessible absorption edge. For long-wavelength phase determination of native structures the choice of wavelength is less clear because the anomalous signal has to be measured at some distance from the absorption edge. Experiments on existing beamlines have suggested an optimal wavelength of around 2.1 Å (Mueller-Dieckmann *et al.*, 2005 ▸).

From a theoretical point of view, the optimal wavelength is dependent on sample size. For smaller samples, longer wavelengths become more favourable in the absence of radiation damage because the increase in the elastic scattering cross-section outweighs the losses owing to absorption (Rosenbaum & Holmes, 1980 ▸; Teplyakov *et al.*, 1998 ▸). Taking radiation damage into account, shorter wavelengths result in more efficient diffraction experiments (Arndt, 1984 ▸). To calculate the optimal wavelength for native SAD experiments, the increase in the anomalous signal towards the sulfur and phosphorus edges needs to be taken into account.

Photo-absorption cross-sections from atoms generally increase roughly as the cube of the wavelength when far from an absorption edge, although less rapidly when closer to the edge. Both the linear absorption coefficient μ_l_ and the imaginary contribution to the scattering factor Δ*f*′′ are proportional to the absorption cross-section (equations 2 and 3). Arndt (1984 ▸) gives an estimate for the variation of the integrated intensity of a Bragg reflection with wavelength in the presence of radiation damage assuming a fixed radiation dose, 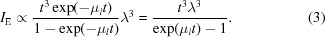
Here, *t* is a linear dimension characterizing the size of an isometric crystal. The equation is based on the assumption that X-ray-induced radiation damage is proportional to the absorbed dose over the wavelength range under consideration (Owen *et al.*, 2006 ▸). In Fig. 1 ▸(*c*) the Bragg intensity *I*_E_ is plotted against wavelength for lysozyme crystals of different sizes and normalized to its value at λ = 2 Å. It can be seen that the diffraction experiment is less efficient for longer wavelengths and is heavily dependent on crystal size.

For native phasing experiments, the intensity differences (Δ*I*_E_) arising from an anomalous substructure rather than the intensities (*I*_E_) should be optimized. An estimate for the Bijvoet differences has been presented by Hendrickson & Teeter (1981 ▸),
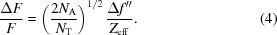

*N*_T_ and *N*_A_ are the total numbers of atoms and of anomalous scatterers, respectively. *Z*_eff_ is the mean number of electrons in protein non-H atoms, which typically has a value of approximately 6.7. Δ*I*/*I* is proportional to Δ*f*′′ since Δ*I*/*I* = 2Δ*F*/*F*. Taking the product of Arndt’s formula (3) and Hendrickson and Teeter’s formula (4),[Disp-formula fd4] it is possible to obtain a prediction of the size of Bijvoet differences. This can be interpreted as a measure of the efficiency of an anomalous phasing experiment taking radiation damage into account,
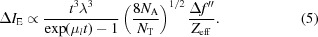
Fig. 1 ▸(*d*) shows plots of (5) for lysozyme crystals of different sizes. To allow comparison, the curves are all normalized to the value at a wavelength of 2 Å.

In summary, for native anomalous phasing experiments a similar crystal-size dependency as for the Bragg intensity (*I*_E_) is observed. However, while for the Bragg intensity short wavelengths are the best choice (Fig. 1 ▸ *c*), when measuring Bijvoet differences for samples smaller than 200 µm the optimal wavelength is shifted to wavelengths beyond 2 Å (Fig. 1 ▸ *d*). In practice, other absorbing but noncrystalline materials surrounding the crystal and the detector efficiency will qualitatively shift the peaks of the curves to shorter wavelengths. However, for crystal sizes smaller than 50 µm, as are nowadays typical for samples studied at third-generation synchrotrons, wavelengths beyond the range offered by standard MX beamlines will be beneficial.

### 2.3. Increased diffraction angles   

**Figure 2:**
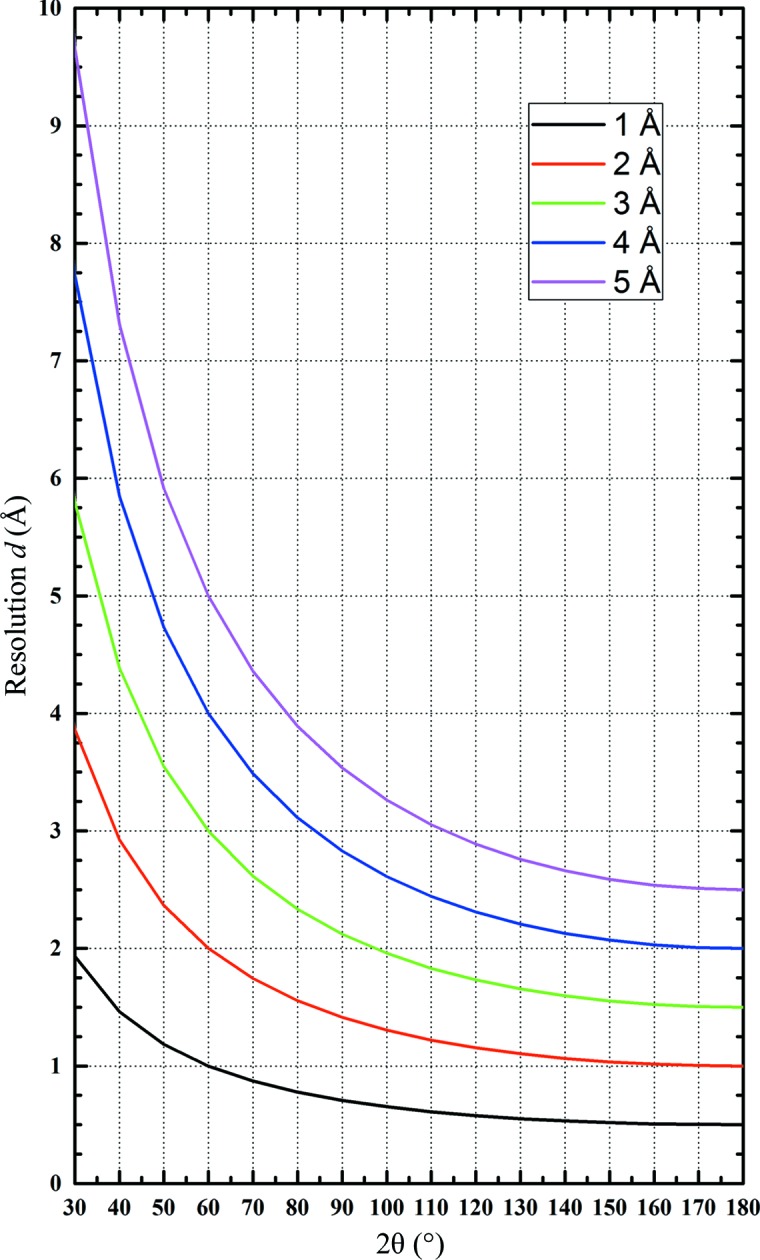
Resolution as function of Bragg angle for different wavelengths.

A major limitation when considering experiments at very long wavelengths are the increased diffraction angles as expressed by Bragg’s law. Fig. 2 ▸ shows the resolution (*d* spacing) as a function of diffraction angle for different wavelengths. To achieve a resolution of at least *d* = 3 Å, it is clear that for wavelengths in the region of λ = 4 Å diffraction angles of larger than 2θ = 90° have to be accessible. In small-molecule crystallography this is typically realised by collecting several sweeps of data at different detector 2θ offsets. Owing to radiation damage, this approach is not ideal in macromolecular crystallography. Therefore, a detector array covering a large range of diffraction angles surrounding the sample is required. Ideally this array should be spherical, but geometrical aspects such as space requirements for the goniometer and sample changer favour a cylindrical arrangement of flat modules. This can be easily achieved by pixel-array detectors, which are typically built from rectangular modules.

### 2.4. Data-collection strategies   

For a cylindrical detector geometry, the blind region for a single sweep around the ω axis of the goniometer increases significantly towards long wavelengths (Dauter, 1999 ▸). Additionally, a cylindrical detector shape yields an unfavourable aspect ratio and hence an uneven coverage of reciprocal space. Therefore, complete data from crystals in low-symmetry space groups are only attainable with a multi-axis goniometer, which allows reorientation of the crystal. As experiments are limited by radiation damage, it is important to get the best out of a given sample. Geometrical data-collection strategies are being developed, taking the beamline geometry, shadowing and collisions into account, to optimize the measurement of anomalous differences for either SAD or MAD experiments.

### 2.5. Optimize *I*/σ   

To measure the anomalous differences accurately, it is important to reduce all sources of noise and instrument error. Performing the experiments in vacuum eliminates scattering from the air path to the crystal between the crystal and the beamstop, as demonstrated by Perutz in 1946 on dried protein crystals at room temperature (Perutz & Rogers, 1946 ▸). The in-vacuum sample environment, in combination with the single-photon-counting detector technology (Broennimann *et al.*, 2006 ▸), reduces the measured background to X-rays scattered only from the crystal and its mount. The optical design of the beamline provides a homogeneous beam illuminating the complete crystal volume and mount. Bathing the crystal in the X-ray beam will keep the diffracting volume constant during the experiment. This will facilitate absorption corrections to compensate for the attenuation of reflections of different path lengths depending on the crystal morphology and orientation. Additionally, crystal vibrations induced by the cryostream, which have been demonstrated to be a major source of instrument error (Alkire *et al.*, 2008 ▸; Flot *et al.*, 2006 ▸), are eliminated by adopting a conductive cooling concept in vacuum.

### 2.6. Absorption corrections   

As already discussed in previous sections, absorption is a limiting factor in long-wavelength MX. Absorption not only contributes to radiation damage, it also attenuates the diffracted X-rays within the sample. Hence, all measured intensities have to be corrected depending on the path lengths that the X-rays travel through the crystal and its surrounding material. Empirical absorption corrections can be performed in the post-refinement step. They are typically based on the refinement of spherical harmonics using intensities from symmetry-related reflections to model the absorption surface (Blessing, 1995 ▸). Therefore, in low-symmetry space groups the applicability of empirical absorption corrections is limited.

X-ray tomography from macromolecular crystals can be used to reconstruct the shape and volume of both diffracting and nondiffracting material in the beamline coordinate system (Brockhauser *et al.*, 2008 ▸). The long-wavelength beamline has an X-ray tomography camera integrated into the design of the endstation. The resulting three-dimensional model will allow the calculation of the path lengths for all measured reflections as the basis for an analytical absorption correction.

Crystal preparation and mounting are important since minimizing the nondiffracting materials surrounding the crystals will reduce absorption effects.

## 3. The in-vacuum long-wavelength MX beamline I23   

### 3.1. Vacuum level   

For wavelengths of up to 4 Å, air absorption and scattering effects are negligible at pressures below 100 Pa. However, the necessity of maintaining protein samples at cryogenic temperatures imposes more rigorous vacuum requirements. The main reason for this is the cryopumping effect: residual gases condense on the surface of a cold object in a vacuum, gradually building up layers of adsorbed molecules. This increasing layer will cause deterioration of the data quality as a result of the absorption and scattering of X-rays. Furthermore, condensation on the cold surfaces of the cryogenic cooling system increases the absorption of thermal radiation, thus reducing the cooling efficiency and causing a gradual warming of all of the components of the system, including the protein crystals. A conservative estimate, assuming free molecular flow and unit sticking probability, determined a target pressure of 10^−4^ Pa for the I23 sample environment. With water vapour considered as the main residual in the vacuum environment, this model suggests an ice-growth rate of about 1 µm over 2.5 h.

### 3.2. Conductive cooling   

Protein crystals dry out quickly when directly exposed to air, a process which is dramatically accelerated in vacuum. Therefore, samples need to be kept at cryogenic temperatures during sample transfer and experiments in vacuum. Most MX experiments are performed at around 100 K to mitigate the problem of radiation damage (Garman, 1999 ▸). It has been shown that a further reduction of temperature to below 100 K is beneficial as it decreases the rate of global damage and specific damage to disulfide bonds (Meents *et al.*, 2010 ▸). Cooling in a vacuum environment relies exclusively upon thermal conductance. A pulse-tube cooler with a base temperature of 8 K is attached to the vacuum vessel. Crystals are cooled through a series of copper links providing a conductive path to the cooler. Flexible links allow rotation around the three angular degrees of freedom of the kappa goniometer. The target temperature at the goniometer head is 30 K, allowing a temperature rise along the cooling path of 22 K. A systematic study was carried out to gain insight into the process of thermal conductance through a system comprised of several materials and interfaces. This included measurements of the thermal contact conductance of breakable copper–copper joints and the conductivity of ice over the temperature range 20–100 K (Mykhaylyk *et al.*, 2012 ▸; Mykhaylyk & Wagner, 2013 ▸). The results guided the development of sample holders and the goniometer head. Neodymium magnets provide the necessary contact force of 12 N required for a good breakable thermal interface.

The sample mount is the most critical part of the sample-holder assembly. While it has to be mechanically robust to support crystals, it must also permit the efficient removal of heat from the sample without causing significant X-ray absorption and scattering. Finite-element analysis suggests that a 10 µm thick sample mount should have a thermal conductivity in excess of 5 W m^−1^ K^−1^ to keep the temperature rise across the sample mount below 20 K during the X-ray diffraction experiment.

Initial experiments were carried out with sample mounts made of metal wires and the electrically conductive polymer DuPont Kapton 50FPC. Metal loops provide efficient cooling but are not compatible with long-wavelength MX because of their high X-ray absorption. The thermal conductance of the DuPont polymer mounts turned out to be insufficient, leading to excessive warming of the protein samples. A number of materials were examined for their thermal conductivity and suitability for diffraction experiments. It was concluded that carbon-based films, such as diamond-like carbon (DLC), chemically vapour-deposited (CVD) diamond or glassy carbon, satisfy these requirements. At this stage the most promising is the glassy carbon Sigradur K, which has better mechanical properties compared with the other materials and has been used in neutron diffraction experiments (Romoli *et al.*, 2014 ▸). Further developments are needed to optimize its use for long-wavelength in-vacuum MX experiments.

### 3.3. Sample transfer   

**Figure 3:**
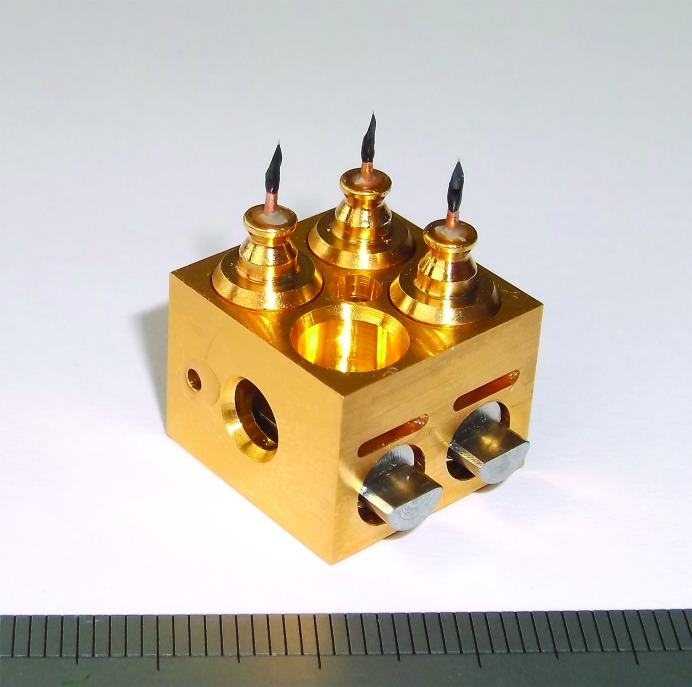
Three sample holders in a copper transfer block. One position is empty.

The transfer of crystals from liquid nitrogen to the I23 in-vacuum endstation relies on an adapted cryo-transfer system (Leica VCT100) designed for sample transfer in cryo-electron microscopy. This system comprises a loading station, a shuttle and a docking station which is attached to the I23 vacuum vessel. Up to four sample holders placed on a copper sample block can be transferred at a time from the liquid-nitrogen bath of the loading station into the cold transfer shuttle. The docking station acts as a load-lock to allow the safe passage of the copper blocks from the shuttle into the sample hotel inside the vacuum vessel. The sample hotel can accommodate a total of 20 crystals on five blocks. A gripper mechanism transfers samples between the hotel and the goniometer. All stages of the sample-loading process are monitored by a machine vision system. A sample block with three sample holders and one empty position can be seen in Fig. 3 ▸.

### 3.4. Beamline layout   

The main beamline components are as follows. X-rays are produced by a 2 m U27 in-vacuum undulator. A double-crystal monochromator (DCM) with two silicon crystals (111 cut) can deliver wavelengths up to 5.9 Å. Two pairs of horizontally deflecting mirrors are used for focusing and harmonic rejection. The first pair of mirrors focuses the beam sagittally and tangentially, while the two flat mirrors of the second pair are used to reject higher harmonics passing the DCM, which is of particular importance at longer wavelengths. The beam can be focused to a modest size of around 120 × 120 µm. This will allow crystals with dimensions of up to 80 µm to be homogeneously illuminated to simplify absorption corrections. Smaller beam sizes can be provided by slits close to the sample. The overall beamline wavelength range is 1–5.9 Å, optimized for experiments in the range from 1.2 to 4 Å.

To avoid the attenuation of long-wavelength X-rays, the beamline is windowless. This means that the endstation, including the sample environment and detector, is directly joined to the storage-ring vacuum, which is realised through differential pumping along the different beamline sections.

### 3.5. Goniometer   

**Figure 4:**
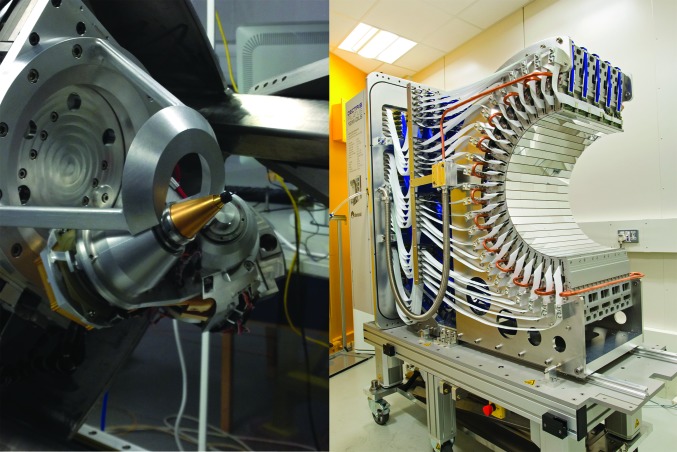
The kappa goniometer during commissioning (left) and the Pilatus 12M before installation (right).

A multi-axis goniometer in inverse-kappa geometry (Brockhauser *et al.*, 2011 ▸) is currently being constructed (Fig. 4 ▸, left) by the UK Astronomy Technology Centre (UK ATC). With an α angle of 50°, it will be possible to align crystals along any given crystallographic axis. Alignment along evenfold axes allows the measurement of Friedel pairs on the same diffraction image with the same absorbed X-ray dose. This can reduce radiation-damage effects in the case of experimental phasing experiments, as both structure factors contributing to an anomalous difference are affected equally. In contrast to conventional kappa goniometers, a conductive cooling link keeps samples at temperatures below 50 K. To allow operation of the beamline during the construction of the mechanically complex kappa goniometer, a single-axis gonio­meter has been installed and is currently used.

### 3.6. Detector   

The I23 beamline is equipped with a large curved Pilatus 12M area detector (Fig. 4 ▸, right). At the start of the project no suitable detector technology for in-vacuum long-wavelength crystallography was available. In collaboration with Dectris, a series of experiments was performed to evaluate the existing Pilatus pixel-array technology (Broennimann *et al.*, 2006 ▸) under these conditions (Marchal & Wagner, 2011 ▸; Marchal *et al.*, 2011 ▸). More recent quantitative work has characterized the quantum efficiency of the Pilatus technology for the detection of long-wavelength X-rays in vacuum even beyond the I23 wavelength range down to λ = 7 Å (Wernecke *et al.*, 2014 ▸).

The Pilatus 12M detector consists of 120 Pilatus2 100k modules arranged in a semi-cylindrical geometry of 24 banks of five modules with a radius of 250 mm. It covers an angular range from 2θ = 40.3° along the equator (cylinder axis) to 2θ = ±100° along the central meridian. The modules are water-cooled to 10°C and can be operated at pressures below 10^−4^ Pa. All modules have been calibrated at long wavelengths and an additional set of ultralow gain settings has been provided by Dectris for operation at longer wavelengths.

## 4. Materials and methods   

### 4.1. Crystallization and sample preparation   

Crystals were obtained by the sitting-drop vapour-diffusion method at 19°C. 5 µl crystallization drops were set up by mixing equal amounts of a 40 mg ml^−1^ solution of thaumatin (Sigma–Aldrich, catalogue No. T7638) in DTNB-saturated deionized water with crystallization buffer consisting of 0.5 *M* potassium/sodium tartrate, 0.05 *M* ADA pH 6.8, 20% glycerol. Crystals were harvested directly from crystallization drops using thermally conductive DuPont Kapton mounts and dipped into a drop of NVH oil (MiTeGen) before being plunge-frozen in liquid nitrogen.

### 4.2. Data collection and processing   

A thaumatin crystal (approximately 200 × 70 × 70 µm) was exposed to X-rays of 1.378 Å wavelength and data were collected on the Pilatus 12M detector with a rotation increment of 0.1° and an exposure of 0.1 s. Diffraction data were integrated with *XDS* (Kabsch, 2010 ▸) and scaled with *AIMLESS* (Evans & Murshudov, 2013 ▸). The heavy-atom substructure was determined with *SHELXD* (Sheldrick, 2010 ▸) and phasing was performed with *autoSHARP* (Vonrhein *et al.*, 2007 ▸). Automatic model building was performed by *ARP*/*wARP* (Langer *et al.*, 2008 ▸) and completed with manual building in *Coot* (Emsley *et al.*, 2010 ▸). *PHENIX* (Adams *et al.*, 2010 ▸) was used for refinement and the secondary-structural alignment was performed with *SUPERPOSE* in *CCP*4 (Krissinel *et al.*, 2004 ▸). The anomalous difference Fourier map was generated with *ANODE* (Thorn & Sheldrick, 2011 ▸).

## 5. Results   

**Figure 5:**
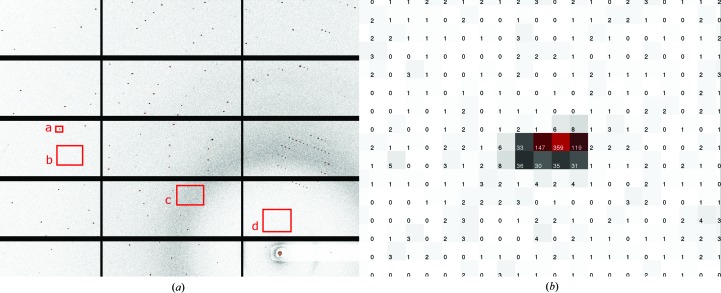
Thaumatin diffraction image (*a*); the boxes are explained in the text. (*b*) is an enlarged image of box a.

An X-ray diffraction pattern collected from the thaumatin crystal is presented in Fig. 5 ▸(*a*). For clarity, only 15 of the 120 detector modules are shown. As a result of placing the entire sample environment and detector in vacuum, the X-ray background is very low. A typical Bragg spot (box a) and its surrounding background are shown in Fig. 5 ▸(*b*). The background is dominated by scattering in the range from zero to two counts. The low background is also manifested as an average of 1.2 counts at medium resolution (Fig. 5 ▸ *a*, box b), 4.4 counts in the solvent ring (box c) and 0.8 counts at low resolution (box d). While these data were collected using a relatively weak unfocused X-ray beam, it is this reduction of noise which yields a high signal-to-noise ratio.

A total of 180° of diffraction data were collected from the crystal. The outstanding data quality allowed successful structure solution with only 90° of data. The high signal-to-noise ratio is reflected in the high value of the asymptotic *I*/σ(*I*) (ISa) of 65.7 (Diederichs, 2010 ▸). Data-processing statistics are listed in Table 1 ▸.

**Figure 6:**
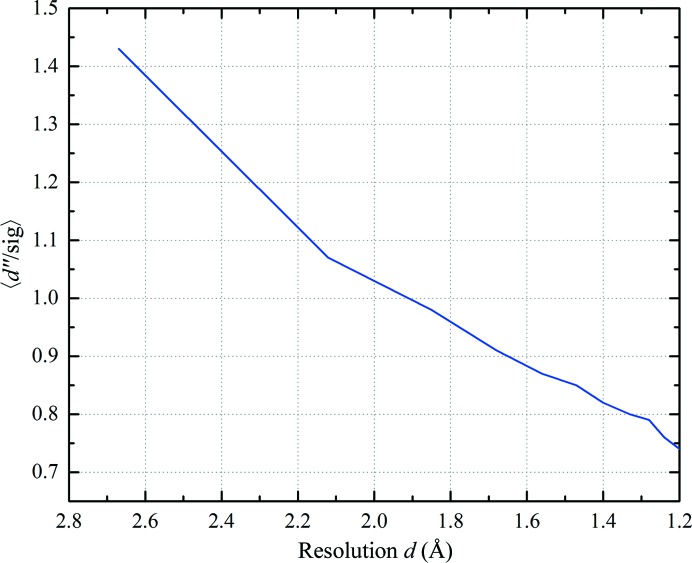
Strength of the anomalous signal Δ*F*/σ(Δ*F*) against resolution from *SHELXC*.

**Figure 7:**
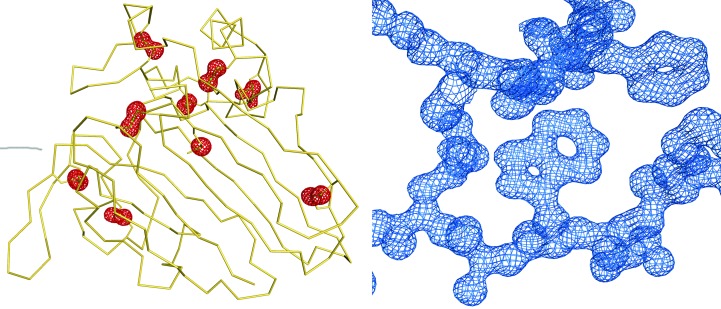
Electron density (2*F*_o_ − *F*_c_) for a fragment of the thaumatin structure (PDB entry 4zg3) contoured at the 1σ level (right). Representation of the C^α^ backbone of thaumatin (yellow) showing the positions of the disulfide bridges (green) with corresponding peaks in the anomalous difference Fourier map (red), contoured at the 4.0σ level (left). One of the disulfide bridges shows disorder.

The Bijvoet ratio for thaumatin at a wavelength of 1.378 Å, as calculated from (4), is 0.98% based on 17 S atoms in 207 amino acids. The strength of the anomalous signal plotted against resolution shell, as estimated by *SHELXC*, is displayed in Fig. 6 ▸. Based on this estimate, a resolution cutoff of 1.5 Å was applied for the subsequent substructure determination. This anomalous signal was sufficient to enable heavy-atom substructure solution and the resulting electron-density map obtained from *autoSHARP* was of excellent quality (Fig. 7 ▸, right), allowing automatic model tracing of 200 of the 207 thaumatin residues. The eight disulfide bridges and the methionine sulfur are clearly visible in the anomalous difference Fourier map (Fig. 7 ▸, left).

The structure of thaumatin solved in vacuum presents no significant differences from other thaumatin structures in the Protein Data Bank. A secondary-structure alignment with three other thaumatin structures determined at comparable resolutions from crystals grown under similar conditions reveals close similarity, in particular to PDB entry 4axr (Cipriani *et al.*, 2012 ▸). The r.m.s.d. value resulting from the superposition of all 207 C^α^ atoms of the two structures is 0.078 Å. The results of the structural alignments are shown in Table 2 ▸, along with the corresponding refinement statistics for all compared structures.

## 6. Conclusion and outlook   

We have presented the first successful native S-SAD structure determination from an in-vacuum MX experiment. The data presented demonstrate that by reducing the background scattering in vacuum in combination with a detector technology without readout noise, very high signal-to-noise ratios can be obtained from low-dose experiments with relatively low multiplicity. While radiation damage will always be a limiting factor, the noise or background reduction will help to manage it and allow more data to be obtained for the same dose compared with standard beamline setups in air.

This study proves that the concept of the in-vacuum long-wavelength MX beamline I23 at Diamond Light Source works. The presented data are very early beamline-commissioning results. After a few modifications to the sample-transfer mechanism, the next step will be to exploit the unique opportunities of this first fully tuneable MX beamline optimized in the wavelength range from λ = 1.2 to 4.0 Å.

## Supplementary Material

PDB reference: thaumatin, 4zg3

## Figures and Tables

**Table 1:** Data-collection and refinement statistics

Data-collection statistics	
Space group	*P*4_1_2_1_2
Unit-cell parameters (Å)	*a* = *b* = 57.84, *c* = 150.37, α = β = γ = 90
X-ray source	I23, DLS
Wavelength (Å)	1.378
Resolution range (Å)	150.37–1.20 (1.22–1.20)
*R*_merge_ (%)	3.7 (76.0)
*R*_p.i.m._ (%)	1.5 (32.9)
Completeness (%)	89.9 (79.9)
Multiplicity	6.8 (5.9)
〈*I*/σ(*I*)〉	26.7 (2.2)
CC_1/2_ (%)	100 (74.4)
ISa	65.70
Mosaicity (°)	0.047
Wilson *B* factor (Å^2^)	8.6
Refinement statistics	
*R*_work_ (%)	14.67
*R*_free_ (%)	16.75
No. of amino-acid residues	207
No. of waters	206
No. of ligands	4
No. of ions	6
R.m.s.d., bond lengths (Å)	0.006
R.m.s.d., angles (°)	1.174
Average *B* factor (Å^2^)	
Protein	15.05
Water	23.77
Ligands	25.77
Ions	29.86
Ramachandran plot	
Favoured (%)	99.1
Allowed (%)	0.9
Outliers (%)	0
Poor rotamers (%)	0.6
Clashscore	2.73
*MolProbity* score	1.06
PDB code	4zg3

**Table 2:** Comparison of the in-vacuum thaumatin structure (PDB entry 4zg3) with three published structures of similar resolution and quality

PDB code	High resolution (Å)	R.m.s.d. (Å)	Refinement *R*_work_ (%)	Refinement *R*_free_ (%)	Unit-cell parameters (Å)
4zg3 (this work)	1.2	—	14.67	16.75	*a* = *b* = 57.84, *c* = 150.37
1lxz (Charron *et al.*, 2002 ▸)	1.25	0.27	17.90	19.50	*a* = *b* = 57.80, *c* = 149.96
3aok (Masuda *et al.*, 2011 ▸)	1.27	0.198	11.20	14.80	*a* = *b* = 57.77, *c* = 150.13
4axr (Cipriani *et al.*, 2012 ▸)	1.38	0.078	13.44	16.56	*a* = *b* = 57.98, *c* = 150.38
